# Book Review: *Routledge Handbook of Animal Welfare*; Knight et al., Eds.; Routledge: Abingdon, UK; New York, NY, USA, 2023; ISBN: 978-1-032-02206-2 (hbk), 978-1-032-32575-0 (pbk), 978-1-003-18235-1 (ebk)

**DOI:** 10.3390/ani13142271

**Published:** 2023-07-11

**Authors:** David Fraser

**Affiliations:** Animal Welfare Program, University of British Columbia, 2357 Main Mall, Vancouver, BC V6T 1Z4, Canada; dfraser@mail.ubc.ca

This information-dense book, edited by two academics and an expert in law, was designed as a “comprehensive exploration of the rapidly growing fields of animal welfare and law”. It packs a huge amount of information and a wide range of topics into its 507 pages written by 50 authors, many of whom are established authorities.

The book opens with three general chapters on “animal welfare fundamentals”. Here, it becomes clear to the reader that the book is pitched as a high-level overview. In Chapter 1, the eminent veterinarian-academic John Webster uses only eight pages to address “The moral status of animals” by covering such topics as the Five Freedoms, duty of care, sentience and consciousness. (A more analytic approach to animal ethics appears later, in Chapter 26). Next, Donald Broom covers “Animal welfare concepts”, including welfare, health, stress, needs, and affective experience, with similar brevity. And Harry Blokhuis and Isabelle Veissier (well known for the Welfare Quality© project) give a brief but valuable overview of different approaches to assessing animal welfare.

Part II contains nine chapters on animal farming, transport, slaughter and euthanasia, with one chapter on each of poultry, pigs, cattle, fish farming, transport and other topics. Each is a remarkably concise overview of complex topics. For example, the excellent chapter on poultry (by Tina M Widowski and Ana K Rentsch) includes a two-page table listing 45 “key welfare concerns” that arise over laying hens, broiler chickens, broiler breeders, ducks, and turkeys, followed by 1–3 paragraphs on specific topics such as claw removal, feed restriction and forced moulting. The focus is mostly on the concerns that arise from high-density housing and genetic selection for productivity traits. However, brief mention is also given to topics such as predation, parasitism and foot-pad dermatitis which are noted as more common in non-cage systems.

The next two sections continue this high-level coverage by focusing on animals used in non-agricultural settings including science and education, entertainment and commercial fishing (Part III), followed by additional species-specific chapters including canines, felines, equines, companion fish, and marine mammals living in human care (Part IV). As one example, Miriam Zemanova’s chapter, “Non-domesticated terrestrial species”, includes a brief introduction to wildlife rehabilitation, wildlife research, repatriation of endangered species, and the exotic pet trade, all within 10 pages of text.

I especially welcomed Part V which provides an introduction to three emerging topics: climate change, animals in disaster management, and the links between animal welfare and human health. Again, the brevity means that these are very concise introductions to complex topics. For example, the chapter on climate change by philosopher Bob Fischer devotes five paragraphs to species introductions and two paragraphs to population declines, extirpations and extinctions.

While such sweepingly broad coverage means that the book favours breadth ahead of depth, most chapters strike a sensible balance among the possible topics. As one example, Michael Cockram’s chapter on animal transport includes transport by road, rail, sea and air, all in 14 pages. He achieved this by summarizing a huge amount of information in a page-long table that listed the key welfare “outcomes” (heat stress, cold stress, injury, fatigue, hunger, etc.) together with the main risk factors and common methods of assessment. This is followed by (typically) one paragraph on each of the welfare concerns and each of the important factors such as ventilation, journey duration and stocking density. Similarly, Moira Harris’s chapter on commercial fishing uses a two-page table to summarize ten capture methods (bottom trawl, drift nets, hook-and-line, etc.), the target species, and the welfare concerns. This is followed by a paragraph or two on such topics as electrical stunning, spiking, bycatch and ghost fishing. My one suggestion for these chapters would be to add a list of key sources for further reading.

Part VI, on “Animal ethics and law”, takes a different and more variable approach. After a brief overview of animal ethics philosophy (Chapter 26) and animal law (Chapter 27), the section includes six chapters on animal law in specific jurisdictions: Australia, China, India, South Africa, the United States and Europe. Some of the chapters are mostly descriptive, while others include significant advocacy. The chapter on Australia, for example, gives a valuable overview of the jurisdictional division of powers, the role of cruelty offences, duties of care and other topics; then roughly one-third of the text is devoted to reforms that the authors recommend such as the creation of a national “statutory entity” in Australia to coordinate the development of policies and standards, plus a more “transparent and accountable” system for creating standards. Similarly, Debbie Legge’s chapter on Europe describes key legal instruments combined with specific reforms that the author would like to see, such as a ban on non-subsistence hunting and on using horses for meat.

Part VII, called “Social change for animals”, expands the shift toward advocacy. A chapter on “Stakeholder groups and perspectives” discusses the engagement of diverse stakeholders in decision-making and also includes a notable digression on the evolution and neurobiology of consciousness and morality. A chapter on “Animal advocacy and human behavioural change” discusses different approaches and strategies for animal protection campaigns. The final chapter focuses on animal welfare education directed toward children, veterinarians, farmers, transporters, and others.

Who will benefit most from this book? Students, veterinarians, and animal welfare scientists might use it to gain a high-level overview of topics outside their specialty. For example, generalist readers will appreciate the valuable overview of the welfare issues of pigs, sheep and goats by authorities such as Sandra Edwards and Cathy Dwyer who have studied these topics for decades. However, with such condensed coverage of technical subjects and roughly a third of the book focused on animal law and social change, I think advocacy workers are the ideal audience. The book would allow them to identify key issues that merit their attention; it outlines different avenues for social change, and it could connect them with others who are trying to achieve better welfare for animals in different parts of the world.

The hardcover book is priced at USD 224 as of mid 2023, but thanks to donations from three animal-advocacy organizations, the contents are available free for download or online reading at: https://doi.org/10.4324/9781003182351 (accessed on 29 June 2023).

